# Convergent Lower Expression of Redox-Linked Stress-Adaptation and Synaptic-Plasticity Genes in Major Depressive Disorder Across Seven Postmortem dlPFC Cohorts

**DOI:** 10.3390/antiox15070908

**Published:** 2026-07-22

**Authors:** Hubert Klepacki, Michal Ordak, Krystyna Kowalczuk, Justyna Magdalena Hermanowicz, Napoleon Waszkiewicz

**Affiliations:** 1 Department of Psychiatry, Medical University of Bialystok, 15-272 Bialystok, Poland; 2 Centre of Regenerative Medicine, Medical University of Bialystok, 15-044 Bialystok, Poland; 3 Department of Integrated Medical Care, Medical University of Bialystok, 15-096 Bialystok, Poland; 4 Department of Clinical Pharmacy, Medical University of Bialystok, 15-222 Bialystok, Poland

**Keywords:** major depressive disorder, dorsolateral prefrontal cortex, oxidative stress, redox regulation, synaptic plasticity, postmortem transcriptomics, gene expression, cross-cohort analysis

## Abstract

Major depressive disorder (MDD) has been linked to oxidative stress, mitochondrial dysfunction, and impaired neuronal plasticity, but the reproducibility of related transcriptomic alterations across postmortem brain cohorts remains uncertain. We performed a targeted cross-platform analysis of a prespecified 14-gene panel spanning antioxidant defense, mitochondrial-redox regulation, cellular stress responses, neurotrophic signaling, synaptic plasticity, and polyamine metabolism across seven postmortem dorsolateral prefrontal cortex cohorts comprising 146 MDD cases and 179 controls. Primary support required Fisher-combined evidence, Benjamini–Hochberg correction across the panel, and concordant MDD-minus-control direction across all available cohorts. *NPTX2*, *EGR1*, *VGF*, *BDNF*, and *SAT1* met these criteria, with lower expression in MDD. The same five-gene pattern was supported by weighted signed Stouffer analysis, one-stage generalized least-squares models, random-effects meta-analysis, and 200,000 disease-label permutations; none produced at least five genes meeting the complete primary-support criterion (empirical *p* = 5.0 × 10^−6^). The most robust cross-cohort finding was a convergent lower-expression pattern across genes supporting redox-linked stress adaptation, polyamine homeostasis, neurotrophic signaling, activity-dependent transcription, and synaptic plasticity. This pattern suggests impaired molecular capacity for neuronal stress resilience and adaptive plasticity in MDD.

## 1. Introduction

Major depressive disorder (MDD) is increasingly understood not as the consequence of a single neurotransmitter abnormality, but as a disorder involving impaired neuronal adaptation, altered cellular stress regulation, and disrupted cortical circuit plasticity. Converging evidence implicates stress-related synaptic remodeling, altered neurotrophic signaling, disturbed activity-dependent transcription, mitochondrial dysfunction, and redox imbalance in its pathophysiology [1,2,3]. These mechanisms are biologically interconnected because neuronal activity and synaptic remodeling depend on coordinated energetic, mitochondrial, and redox support.

The prefrontal cortex is particularly relevant to these processes because it supports emotion regulation, cognitive control, integration of stress-related signals, and flexible behavioral adaptation. Abnormalities of prefrontal cortical structure and function are among the most frequently reported neurobiological findings in depression, while impairments in cognitive-control circuitry are also observed across psychiatric disorders [4,5,6,7]. Postmortem transcriptomic studies therefore provide an important opportunity to examine molecular alterations directly in cortical regions implicated in MDD.

Disease-related alterations in the dorsolateral prefrontal cortex (dlPFC) may involve interacting disturbances in synaptic function, neurotrophic signaling, activity-dependent transcription, mitochondrial and redox homeostasis, cellular stress responses, polyamine metabolism, and extracellular-matrix remodeling [2,8,9,10]. These processes are unlikely to operate independently. Integrative models suggest that multiple biological systems implicated in MDD may interact and ultimately converge on synaptic function [11]. From this perspective, molecular alterations in MDD may be better understood as disruptions of interconnected stress-, redox-, metabolic-, and plasticity-related pathways rather than as isolated abnormalities in individual genes.

A major challenge in postmortem transcriptomic studies of MDD is limited reproducibility across datasets. Differences in cohort composition, diagnostic procedures, medication exposure, agonal state, postmortem interval, tissue pH, RNA integrity, measurement platform, and preprocessing strategy can introduce substantial biological and technical heterogeneity [12,13,14]. Tissue pH and terminal medical conditions may systematically influence postmortem brain gene-expression profiles [13], while platform-, preprocessing-, and study-level differences complicate the integration of microarray datasets and the interpretation of cross-study findings [12,14]. Consequently, gene-expression signals identified in a single cohort may not represent stable or reproducible MDD-associated alterations.

To address this problem, we prioritized cross-cohort reproducibility using a focused, biologically defined candidate-gene panel. Rather than directly pooling heterogeneous transcriptomic datasets or performing an unrestricted transcriptome-wide discovery analysis, this approach asked whether genes representing predefined biological domains showed directionally consistent expression differences across postmortem dlPFC cohorts. Such a design reduces the multiple-testing burden, limits overinterpretation of isolated findings, and places greater emphasis on reproducible effect direction across studies.

The literature-informed 14-gene panel was structured by biological domain. A synaptic, neurotrophic, and activity-dependent module included *BDNF*, *VGF*, *EGR1*, and *NPTX2*, reflecting prior evidence for altered neuronal plasticity, neurotrophic support, immediate-early gene regulation, and synaptic organization in depression-related phenotypes [15,16,17,18]. A mitochondrial and redox-related module included *GPX4*, *PRDX2*, *PRDX3*, *SOD2*, *MFN1*, and *MTOR*, representing canonical antioxidant defense and peroxide metabolism [1,19], ferroptosis-related lipid-peroxide control [20,21], mitochondrial dynamics [22], and metabolic stress signaling [23]. *ADAMTS16*, *ZNF701*, *SAT1*, and *SKA2* were retained as exploratory candidates related to extracellular-matrix remodeling, transcriptional regulation, polyamine metabolism, and stress-associated molecular phenotypes [10,24,25,26,27,28,29].

The panel was not designed as a diagnostic biomarker set or as a collection of exclusively antioxidant genes. Instead, it was intended to test whether reproducible MDD-associated expression signals occurred within canonical mitochondrial-redox pathways, within stress- and plasticity-related pathways, or across both biological domains.

The primary objective of this study was to determine whether genes from this candidate panel showed reproducible expression differences between individuals with MDD and controls across postmortem dlPFC cohorts. We focused on concordance of effect direction across cohorts and on aggregate statistical evidence within the prespecified panel rather than on dataset-specific significance alone. We hypothesized that the most consistent signal would emerge from interconnected stress-adaptation, neurotrophic, and synaptic-plasticity pathways, consistent with models in which diverse molecular disturbances in MDD converge on impaired synaptic function [2,11].

## 2. Materials and Methods

### 2.1. Study Design and Datasets

This targeted cross-cohort analysis evaluated a prespecified 14-gene panel in postmortem dorsolateral prefrontal cortex tissue across seven analytical cohorts: GSE54567, GSE54568, GSE102556, GSE101521, GSE53987, GSE208338, and GSE213982. Because GSE54567 and GSE54568 originated from the same study, the dataset comprised seven analytical cohorts from six independent projects. Although GSE54567 and GSE54568 originated from the same parent study and were generated on the same GPL570 platform, they comprised distinct matched case–control sample sets and were therefore retained as separate analytical cohorts. They should not, however, be regarded as fully independent study-level or technical replications. The pooled analysis included 325 donors. Donor-level expression estimates from GSE213982 were used for the disease-effect analysis, separately from the cell-type localization analysis.

### 2.2. Candidate Panel

**Figure 1:**
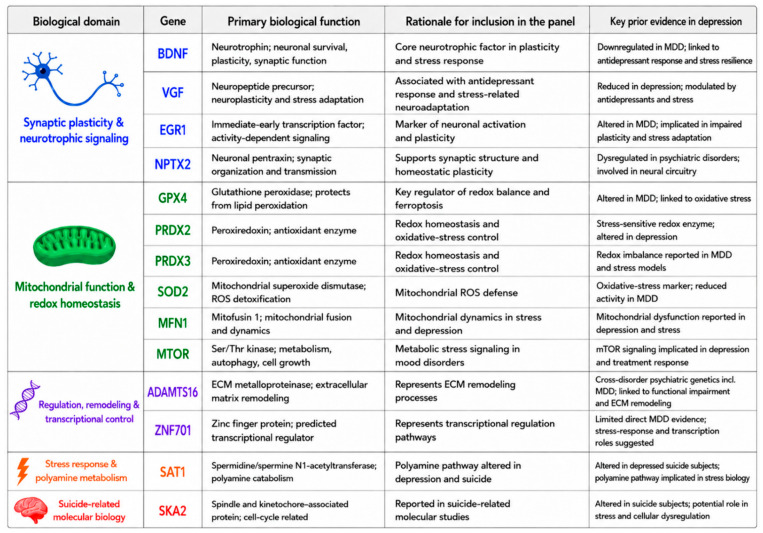
**Biological rationale for the candidate gene panel.** The figure summarizes the 14-gene candidate panel according to broad biological domains relevant to MDD and stress-related phenotypes. Gene selection was domain-based rather than diagnostic; therefore, individual genes differed in the amount of prior direct evidence in human prefrontal cortex MDD studies. The biological rationale and prior evidence summarized in the figure are based on the literature cited in the Introduction. The figure was created with BioRender.com.

The prespecified 14-gene panel included *GPX4* (glutathione peroxidase 4), *PRDX2* (peroxiredoxin 2), *VGF* (VGF nerve growth factor inducible), *ADAMTS16* (ADAM metallopeptidase with thrombospondin type 1 motif 16), *NPTX2* (neuronal pentraxin 2), *EGR1* (early growth response 1), *ZNF701* (zinc finger protein 701), *MFN1* (mitofusin 1), *SOD2* (superoxide dismutase 2), *MTOR* (mechanistic target of rapamycin kinase), *PRDX3* (peroxiredoxin 3), *SKA2* (spindle and kinetochore associated complex subunit 2), *SAT1* (spermidine/spermine N1-acetyltransferase 1), and *BDNF* (brain-derived neurotrophic factor); its literature rationale and biological-domain structure are described in the Introduction and summarized in Figure 1. All genes were retained regardless of statistical significance or direction of effect. The candidate-gene panel was prospectively fixed before primary cross-cohort testing and was not modified on the basis of cohort-specific expression results, nominal *p*-values, Fisher-combined evidence, FDR-adjusted results, or direction patterns. Time-stamped documentation of the fixed panel and its biological rationale is available on OSF: https://osf.io/8bfrs/overview?view_only=6f7fbb64f2da46ada7fd619c72a7d86d (accessed on 30 May 2026).

The panel was intentionally heterogeneous and was not intended to represent a set of core MDD genes only; rather, it was designed as a broader stress-adaptation panel spanning synaptic and neurotrophic plasticity, mitochondrial-redox biology, polyamine metabolism, extracellular-matrix remodeling, transcriptional regulation, and stress-associated molecular phenotypes. Accordingly, *ADAMTS16* and *ZNF701* were included as exploratory remodeling/transcriptional-regulatory candidates with more indirect prior relevance to MDD than *BDNF*, *VGF*, *EGR1*, and *NPTX2*.

### 2.3. Expression Extraction and Preprocessing

For GSE54567 and GSE54568, expression values were obtained from GEO series matrix files, and diagnostic groups were assigned from public sample annotations. Because both GPL570 cohorts had matched case–control designs, effects were estimated as within-pair MDD-minus-control differences and tested using paired *t*-tests. One representative probe set per gene was fixed before cross-cohort testing based on direct HGNC-symbol annotation and availability in both cohorts. Probe-selection criteria, the frozen probe map, and probe-level sensitivity analyses are provided in Supplementary Tables S1 and S2.

For GSE102556, BA8/9 RNA-seq expression was analyzed as log2(FPKM + 1), whereas GSE101521 was analyzed as log2(normalized count + 1). GSE53987 and GSE208338 were analyzed using their deposited processed expression values; in GSE208338, gene-level expression was defined as the median across available eligible probe sets. Donor-level gene-expression estimates were used for GSE213982. Unpaired cohorts were tested using Welch’s two-sample *t*-tests.

Because platforms and preprocessing scales differed, expression values were analyzed within cohorts rather than pooled directly. For each gene, the cohort-specific effect was defined as the unstandardized MDD-minus-control mean difference, with negative values indicating lower expression in MDD.

### 2.4. Primary Cross-Cohort Analysis

Because platforms and preprocessing scales differed, the primary analysis combined cohort-level nominal *p*-values rather than pooling expression values. Fisher’s method was used to integrate evidence across cohorts, followed by Benjamini–Hochberg correction across the prespecified 14-gene panel. Primary support required both BH-FDR < 0.05 and a concordant MDD-minus-control direction across all available cohorts. Genes with significant combined *p*-values but discordant directions were not classified as primary-support genes.

### 2.5. Sensitivity Analyses

Sensitivity analyses included a sample-size-weighted signed Stouffer combination, with Benjamini–Hochberg correction across the prespecified 14-gene panel. Cohort weights were based on the number of matched pairs for paired cohorts and the effective sample size for unpaired cohorts. Distributional assumptions were assessed using Shapiro–Wilk tests.

A one-stage generalized least-squares model was fitted to expression values standardized within each gene and cohort. The model estimated a common diagnosis effect using cohort-specific intercepts and covariance blocks to account for the matched case–control design of GSE54567 and GSE54568. Diagnosis-by-cohort interaction terms were used to assess variation in effect magnitude across cohorts.

A complementary random-effects meta-analysis was performed using standardized mean differences. For the unpaired cohorts, effect sizes were calculated as conventional Hedges’ g using the pooled within-group standard deviation. The small-sample correction was based on nMDD + ncontrol − 2 degrees of freedom. For GSE54567 and GSE54568, which used matched case–control designs, effect sizes were calculated as Hedges’ gav. The MDD-minus-control mean difference was standardized by the square root of the average of the two group variances, allowing paired and unpaired effects to be expressed on a comparable outcome-standard-deviation scale. The small-sample correction was based on npairs − 1 degrees of freedom, and the sampling variance incorporated the observed within-pair correlation, following Morris and DeShon [30].

Between-cohort variance was estimated using restricted maximum likelihood, and pooled effects were calculated using inverse-variance weighting. Heterogeneity was summarized using Cochran’s Q, tau-squared, and I-squared. Confidence intervals and two-sided *p*-values were based on conventional normal-theory Wald inference, with 95% confidence intervals calculated as the pooled estimate ± 1.96 standard errors. Benjamini–Hochberg correction was applied across the prespecified 14-gene panel. All sensitivity analyses were considered complementary and did not redefine primary support.

An exploratory diagnosis-by-sex analysis was performed across all seven cohorts using a one-stage generalized least-squares model with cohort-specific intercepts and terms for diagnosis, sex, and diagnosis-by-sex interaction. Expression was standardized within each gene and cohort, female sex was used as the reference category, and covariance blocks accounted for the matched designs of GSE54567 and GSE54568. Benjamini–Hochberg correction was applied across the prespecified 14-gene panel; this analysis was considered exploratory and did not redefine primary support. Full results are provided in Table S2.

All statistical analyses were performed in Python 3.13.5 using NumPy 2.3.5, pandas 2.2.3, and SciPy 1.17.0. Figures were generated using Matplotlib 3.10.8. Supplementary File S1 contains the complete analysis code, package requirements, processed input data, validation scripts, and instructions for reproducing the reported results.

### 2.6. Permutation-Based Sensitivity Analysis

Disease labels were permuted within each cohort while preserving the original study design. For GSE54567 and GSE54568, case–control status was randomly swapped within matched pairs. For the remaining cohorts, diagnosis labels were permuted while preserving group sizes. The same permuted labels were applied to all genes within each cohort to retain the within-cohort correlation structure.

For each of 200,000 permutations, the complete primary pipeline was repeated, including cohort-level testing, Fisher combination, Benjamini–Hochberg correction across the 14-gene panel, and assessment of concordant direction across all available cohorts. The empirical panel-level *p*-value was defined as the proportion of permutations producing at least as many primary-support genes as observed, using add-one correction.

### 2.7. Single-Nucleus RNA-Seq Cell-Type Localization

In addition to its donor-level disease-effect analysis, GSE213982 was used as a descriptive cell-type localization resource. Candidate-gene counts from the deposited combined dlPFC single-nucleus RNA-seq matrix were summarized across major annotated cell classes, including excitatory and inhibitory neurons, astrocytes, oligodendrocytes, oligodendrocyte precursor cells, microglia, and endothelial cells. For each gene, the neuronal proportion was calculated as:

100 × (excitatory-neuron counts + inhibitory-neuron counts)/total observed counts.

This analysis was used only to contextualize the cellular localization of candidate genes and was not interpreted as cell-type-specific differential expression.

### 2.8. Analytical Interpretation

Primary support required combined evidence from Fisher’s method, BH-FDR < 0.05 across the 14-gene panel, and a concordant MDD-minus-control direction across all available cohorts. Sample-size-weighted signed Stouffer analysis, one-stage GLS, random-effects meta-analysis, permutation testing, and cell-type localization were treated as sensitivity or contextual analyses and did not redefine primary support. Supported genes were interpreted as cross-cohort expression signals rather than diagnostic biomarkers or causal mechanisms.

## 3. Results

### 3.1. Dataset Overview

The primary analysis included seven cohort-level contrasts from six independent public postmortem dlPFC studies, comprising 146 MDD cases and 179 controls across 325 donors. Dataset-level characteristics are summarized in Table 1.

### 3.2. Primary Cross-Cohort Support

**Figure 2:**
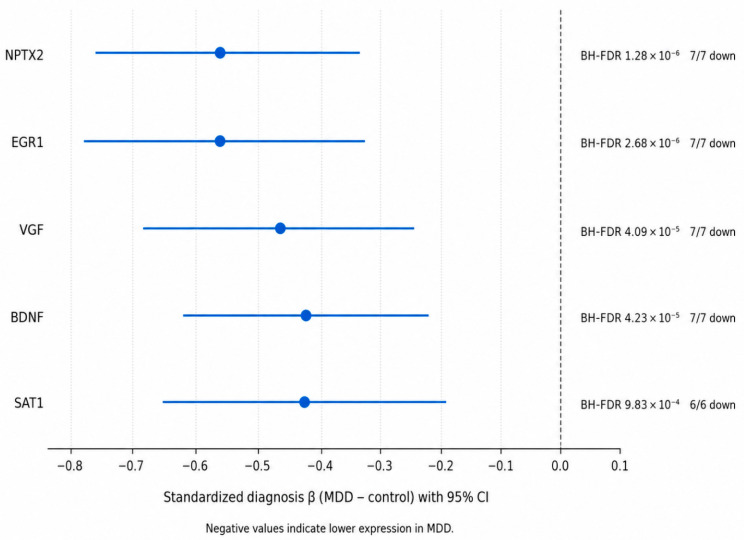
**One-stage generalized least-squares analysis of the five primary-support genes across seven analytical cohorts.** Standardized diagnosis coefficients for MDD relative to controls are shown with 95% confidence intervals. Negative coefficients indicate lower expression in MDD. Right-hand labels report Benjamini–Hochberg-adjusted *p*-values from the GLS analysis and the number of available cohorts showing concordant lower expression in MDD. *SAT1* was analyzed in six cohorts because processed expression values were unavailable in GSE208338.

Five genes met the criteria for primary cross-cohort support, requiring both Fisher BH-FDR < 0.05 and concordant MDD-minus-control direction across all available cohorts: *EGR1*, *NPTX2*, *VGF*, *BDNF*, and *SAT1*. All five showed lower expression in MDD. *EGR1*, *NPTX2*, *VGF*, and *BDNF* were concordantly downregulated across all seven cohorts, whereas *SAT1* showed concordant downregulation across the six cohorts in which processed expression data were available. Standardized diagnosis coefficients from the one-stage generalized least-squares analysis are shown in Figure 2, and full panel-level primary results are presented in Table 2.

Sample-size-weighted signed Stouffer analysis supported the same five primary-support genes after correction across the 14-gene panel: *NPTX2*, *EGR1*, *VGF*, *BDNF*, and *SAT1*. One-stage GLS likewise identified significantly lower expression of all five genes, with BH-FDR values ranging from 1.28 × 10^−6^ to 9.83 × 10^−4^. Diagnosis-by-cohort interactions were not significant after multiple-testing correction. Shapiro–Wilk diagnostics indicated departures from normality in a subset of gene–cohort distributions and model residuals and were interpreted as distributional diagnostics rather than criteria for redefining primary support.

Permutation analysis further supported the fixed-panel result. None of 200,000 disease-label permutations produced at least five genes meeting the complete primary-support criterion of Fisher BH-FDR < 0.05 together with concordant direction across all available cohorts. The add-one-corrected empirical panel-level *p*-value was 5.0 × 10^−6^. Full sensitivity and probe-level results are provided in the Supplementary Tables.

### 3.3. Genes with Partial or Discordant Support

Several candidate genes did not meet the complete primary-support criteria. *GPX4* survived Fisher BH correction but showed discordant effect directions across cohorts and was therefore not classified as supported. *SKA2*, *ADAMTS16*, *PRDX2*, and *SOD2* showed nominal or near-threshold aggregate evidence but also lacked complete directional concordance. The remaining genes did not survive panel-level correction, showed discordant directions, or both. These findings support the joint use of combined statistical evidence and cross-cohort directional concordance.

### 3.4. One-Stage Individual-Level Sensitivity Analysis

All five primary-support genes showed significantly lower expression in MDD in one-stage generalized least-squares models of cohort-standardized individual-level data (Table 3). Standardized diagnosis coefficients ranged from −0.435 to −0.557, and all five genes remained significant after Benjamini–Hochberg correction across the 14-gene panel. No diagnosis-by-cohort interaction remained significant after multiple-testing correction.

### 3.5. Random-Effects Meta-Analysis of Standardized Effects

**Figure 3:**
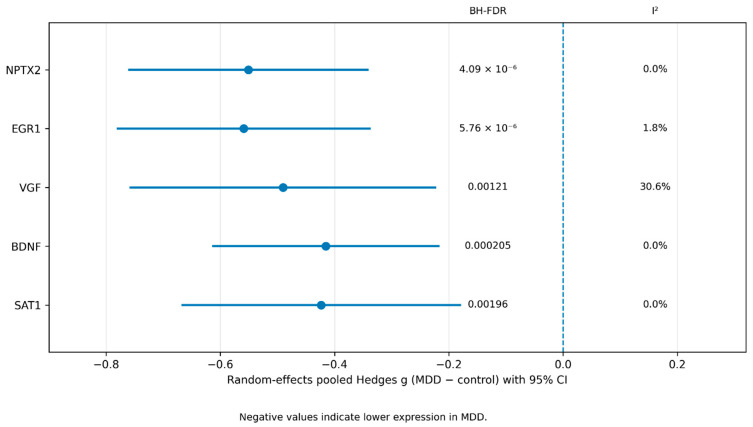
**Random-effects meta-analysis of standardized expression differences for the five primary-support genes.** Pooled Hedges g estimates are shown with 95% confidence intervals. Negative values indicate lower expression in MDD. Random-effects models were estimated using restricted maximum likelihood. Right-hand labels show Benjamini–Hochberg-adjusted *p*-values and I^2^ heterogeneity estimates. *SAT1* included six cohorts because processed expression values were unavailable in GSE208338.

A complementary random-effects meta-analysis of standardized mean differences confirmed significantly lower expression of all five primary-support genes (Figure 3). Pooled Hedges g values were −0.551 for *NPTX2*, −0.559 for *EGR1*, −0.490 for *VGF*, −0.415 for *BDNF*, and −0.423 for *SAT1*, with all 95% confidence intervals excluding zero. All five genes remained significant after Benjamini–Hochberg correction across the 14-gene panel. Between-cohort heterogeneity was negligible for *NPTX2*, *EGR1*, *BDNF*, and *SAT1* (I^2^ = 0–1.8%) and moderate for *VGF* (I^2^ = 30.6%). These findings were consistent with the Fisher, weighted signed Stouffer, and one-stage GLS analyses.

### 3.6. Cell-Type Localization of Candidate Genes in the GSE213982 snRNA-Seq dlPFC Resource

The five primary-support genes showed predominantly neuronal localization in the GSE213982 dlPFC single-nucleus RNA-seq count matrix, with most observed counts assigned to excitatory and inhibitory neuronal nuclei (Table 4). This analysis was used only to provide descriptive cellular context and was not interpreted as an independent validation or as evidence of cell-type-specific differential expression.

## 4. Discussion

### 4.1. Principal Findings

In this targeted cross-cohort analysis of seven postmortem dlPFC cohorts, five genes from the prespecified 14-gene panel—*NPTX2*, *EGR1*, *VGF*, *BDNF*, and *SAT1*—showed reproducibly lower expression in MDD across all available cohorts. The same pattern was supported by complementary cohort-level, sample-level, meta-analytic, and permutation-based analyses, while no diagnosis-by-cohort interaction remained significant after multiple-testing correction. These findings should therefore be interpreted as reproducible candidate-gene expression signals rather than diagnostic biomarkers, causal mechanisms, or a transcriptome-wide molecular profile of MDD.

The principal result was not a uniform alteration of canonical antioxidant enzymes. Instead, the most consistent cross-cohort signal involved genes linking neurotrophic support, activity-dependent transcription, synaptic organization, polyamine metabolism, and redox-sensitive stress adaptation. This pattern is consistent with models in which molecular disturbances in MDD converge on impaired synaptic function and reduced adaptive plasticity rather than on a single isolated biochemical pathway [11,17,31].

### 4.2. A Convergent Neurotrophic, Activity-Dependent, and Synaptic Signature

Four of the five primary-support genes—*BDNF*, *VGF*, *EGR1*, and *NPTX2*—formed a coherent neuronal plasticity-related module. *BDNF* and *VGF* represent neurotrophic and synaptic-plasticity processes, *EGR1* reflects activity-dependent transcriptional regulation, and *NPTX2* contributes to excitatory synaptic organization. Their shared lower-expression direction across all seven cohorts suggests a convergent disturbance of molecular systems required for neuronal adaptation rather than an isolated alteration of a single transcript.

BDNF is a major regulator of neuronal survival, growth, maturation, synaptogenesis, neuroprotection, and activity-dependent synaptic plasticity [32,33]. It also modulates neurotransmitter release, synaptic transmission, and neuronal excitability [32,34]. Both acute and chronic stress can reduce *BDNF* mRNA expression, particularly in the hippocampus, although the magnitude and regional distribution of this effect depend on the nature and duration of the stressor [35,36].

The relationship between BDNF signaling and redox homeostasis may be bidirectional. Stress-related increases in reactive oxygen species and disturbances in cellular antioxidant defenses can interfere with signaling pathways required for neuronal plasticity [3]. In a chronic social defeat stress model, stress susceptibility was associated with lower Nrf2 and BDNF levels in the medial prefrontal cortex and hippocampus, whereas pharmacological activation of Nrf2 increased *BDNF* transcription and improved synaptic abnormalities [37]. Conversely, reduced BDNF signaling may weaken Nrf2-dependent antioxidant responses and promote persistent oxidative stress [38]. Chronic stress, impaired BDNF–TrkB signaling, and excessive ROS production may therefore form a mutually reinforcing system that reduces neuronal plasticity and increases cellular vulnerability.

*VGF*, a neurotrophin-inducible gene functionally linked to BDNF signaling, has also been implicated in synaptic plasticity, neurogenesis, and depression-related phenotypes [15,39]. Administration of the VGF-derived peptide TLQP-62 attenuated LPS-induced memory impairment and anxiety- and depression-like behaviors in mice. These effects were accompanied by reduced neuroinflammatory and oxidative responses and depended, at least partly, on intact BDNF–TrkB signaling [40]. Lower *VGF* expression may therefore be associated with reduced availability of neurotrophic and stress-protective signaling. However, the present data do not establish changes in VGF peptide abundance or demonstrate a direct effect on inflammatory or oxidative pathways.

*EGR1* provides an activity-dependent transcriptional component to the lower-expression signature. As an immediate-early gene, *EGR1* links neuronal activation to downstream programs involved in learning-related adaptation, synaptic plasticity, and stress-responsive remodeling [16]. Acute hydrogen peroxide exposure can rapidly induce *EGR1* expression as part of an immediate-early cellular stress response [41]. In contrast, chronic stress has been associated with reduced *EGR1* expression in prefrontal cortical regions. Lower immediate-early gene expression, including *EGR1*, has been reported in prefrontal cortical tissue from individuals with MDD and in the medial prefrontal cortex of mice exposed to chronic social defeat stress [42]. Consistent with broader involvement of *EGR1* dysregulation in psychiatric disorders, reduced *EGR1* expression has also been reported in peripheral blood from affected patients, although such peripheral findings cannot be directly equated with brain expression [43]. Chronic social isolation likewise reduced *Egr1* expression in the prefrontal cortex and hippocampus and was accompanied by anxiety- and depression-like behaviors [44]. The lower *EGR1* expression observed here may therefore reflect chronic-stage impairment or reorganization of redox-sensitive, activity-dependent transcription rather than the transient induction typically observed following an acute oxidative challenge.

### 4.3. NPTX2, AMPA-Dependent Synaptic Organization, and PV Interneurons

NPTX2/NARP is an activity-regulated neuronal pentraxin involved in AMPA receptor-dependent excitatory synapse organization and in the regulation of excitatory inputs onto parvalbumin-positive interneurons [45,46]. These fast-spiking interneurons contribute to excitatory–inhibitory balance, temporal precision, and synchronization of cortical activity. Lower *NPTX2* expression may therefore indicate weaker molecular support for the excitatory synaptic organization required for effective recruitment of PV interneurons and stable prefrontal network function.

This interpretation is particularly relevant under chronic stress. Chronic stress and glucocorticoid exposure can disrupt glutamatergic transmission, synaptic remodeling, and structural plasticity in stress-sensitive brain regions [47]. Cell-type-specific transcriptomic analyses of the medial prefrontal cortex have further shown that chronic stress disrupts postsynaptic, metabolic, bioenergetic, oxidative-phosphorylation, and oxidative-stress-response programs across pyramidal neurons and inhibitory interneurons, including PV cells [48]. PV interneurons have substantial energetic requirements and are particularly sensitive to disturbances in redox homeostasis; experimental oxidative stress can compromise their structural and functional integrity [49]. Stress susceptibility has also been associated with weakened excitatory synaptic transmission onto PV interneurons in the prefrontal cortex [50].

In this context, lower *NPTX2* expression may represent a synaptic component of stress-related excitatory–inhibitory dysregulation, particularly when chronic stress simultaneously disrupts energy metabolism, redox homeostasis, and excitatory recruitment of PV interneurons. This interpretation remains indirect because the available evidence does not establish that elevated ROS directly causes *NPTX2* downregulation in MDD. Because PV interneurons provide the temporally precise inhibition required for cortical gamma synchronization, impaired *NPTX2*-dependent excitatory recruitment of these cells could promote prefrontal disinhibition and disturb gamma-band coordination. Postmortem evidence links lower NARP expression to reduced markers of PV-interneuron activity in the human dlPFC [51], while human transcranial magnetic stimulation–electroencephalography (TMS–EEG) evidence indicates that GABA*_B_*-mediated cortical inhibition suppresses gamma oscillations in this region [52]. Such circuit-level alterations may be relevant to deficits in working memory and cognitive control, although neither cortical oscillations nor cognitive performance was assessed in the present study.

The involvement of NPTX2 in AMPA receptor-dependent synaptic organization may also be relevant to glutamatergic mechanisms implicated in rapid antidepressant response. Although ketamine and esketamine act primarily as NMDA receptor antagonists, preclinical evidence indicates that their plasticity-related effects cannot be explained by NMDA receptor blockade alone and require intact AMPA receptor-mediated transmission. Pharmacological blockade of AMPA receptors attenuates or abolishes the rapid antidepressant-like effects of ketamine and prevents the associated upregulation of mTOR- and BDNF-related signaling [53,54]. Ketamine has also been shown to rapidly activate mTOR signaling, increase synaptic-protein expression, and promote the formation and functional maturation of new dendritic spine synapses in the prefrontal cortex, whereas inhibition of mTOR blocks both its synaptogenic and behavioral effects [55]. Rapid behavioral responses to ketamine further depend on *de novo* BDNF synthesis [56]. The convergent lower expression of *NPTX2* and *BDNF* observed here may therefore indicate reduced molecular capacity within an AMPA–mTOR–BDNF-related plasticity system. However, the present study did not assess treatment response, and a direct role of NPTX2 in the effects of ketamine or esketamine has not been established. Moreover, most mechanistic evidence for this pathway derives from animal studies of racemic ketamine and should be extrapolated to esketamine with caution.

### 4.4. SAT1 and Polyamine-Related Redox Regulation

*SAT1* extends the primary pattern beyond classical neurotrophic and synaptic mechanisms. It encodes the rate-limiting enzyme of spermidine and spermine acetylation and therefore occupies a central position in intracellular polyamine turnover. Acetylated polyamines may be exported from the cell or further oxidized by acetylpolyamine oxidase, producing hydrogen peroxide and reactive aldehydes [57,58]. At the same time, spermidine and spermine can protect cells against oxidative damage. Consequently, disturbances of SAT1-dependent metabolism may influence redox homeostasis through several potentially opposing mechanisms rather than through a simple linear pathway.

Postmortem RNA-seq evidence from the dlPFC has shown reduced *SAT1* expression in both suicidal and non-suicidal individuals with MDD, suggesting that lower brain *SAT1* expression may be associated with depression itself rather than exclusively with suicidal behavior [28]. More broadly, *SAT1* downregulation and polyamine-system dysregulation have been linked to depression, suicide-associated molecular phenotypes, cellular stress responses, neurotransmission, and activity-dependent plasticity [10,57,58,59,60].

Within the present framework, lower *SAT1* expression may reflect impaired regulation of polyamine turnover and reduced metabolic flexibility under chronic stress. Such dysregulation could alter redox balance while also constraining adaptive synaptic and cellular remodeling in the dlPFC. The broader cellular distribution of *SAT1* in the descriptive single-nucleus analysis further suggests that its contribution may be less exclusively neuronal than that of *BDNF*, *VGF*, *EGR1*, and *NPTX2*.

### 4.5. Canonical Antioxidant, Mitochondrial, and Sex-Dependent Signals

Biological plausibility alone was insufficient for classification as a reproducible cross-cohort signal. *GPX4* survived panel-level correction of the Fisher-combined *p*-value but showed discordant effect directions across cohorts and was therefore not classified as a primary-support gene. *SKA2*, *ADAMTS16*, *PRDX2*, and *SOD2* showed nominal or near-threshold aggregate evidence but lacked complete directional concordance, whereas *PRDX3* showed weaker combined evidence and discordant directions. *MFN1* showed lower expression in six of seven cohorts but likewise failed to meet the prespecified support criteria because it lacked complete directional concordance and significant aggregate statistical evidence.

This distinction guards against selective interpretation of biologically plausible genes. The lack of consistent direction among *GPX4*, *PRDX2*, *PRDX3*, and *SOD2* should be regarded as an informative negative finding rather than merely as an absence of statistical support. Within the present targeted analysis, these canonical antioxidant-enzyme transcripts did not behave as stable cross-cohort MDD-associated expression signals in postmortem dlPFC tissue. The results therefore argue against generalized suppression of canonical antioxidant defenses and instead suggest context-dependent reorganization of redox, mitochondrial, metabolic, and synaptic stress-response pathways.

This heterogeneity does not argue against oxidative stress or redox dysregulation in MDD. Antioxidant transcriptional responses may differ according to the intensity, duration, and cellular context of stress. Mild acute oxidative stress can activate compensatory NRF2-dependent cytoprotective transcription in brain-resident cells; in astrocytes, H_2_O_2_ exposure induced NRF2-dependent antioxidant and stress-response gene expression [61]. In contrast, prolonged or repeated stress may be accompanied by an insufficient or maladaptive NRF2 response. In a stress-vulnerability model, low BDNF levels in susceptible animals impaired NRF2 nuclear translocation and prevented activation of detoxifying and antioxidant enzymes, resulting in persistent oxidative stress [38]. Increased, decreased, or unchanged expression of individual antioxidant enzymes may therefore reflect different stages of compensation, adaptation, or response failure.

The more reproducible pattern identified here involved genes connecting redox-sensitive stress adaptation with polyamine homeostasis, neurotrophic signaling, activity-dependent transcription, and synaptic organization. The convergent lower expression of *EGR1*, *NPTX2*, *VGF*, *BDNF*, and *SAT1* may represent a more stable correlate of impaired long-term cellular adaptation than the context-dependent expression of individual antioxidant enzymes. Future studies should integrate antioxidant-gene expression with direct measurements of NRF2/ARE activity, glutathione status, lipid peroxidation, and antioxidant-enzyme activity. Future studies should integrate these direct redox measurements with downstream stress-adaptation, polyamine, and neuroplasticity pathways rather than evaluating isolated antioxidant transcripts alone.

Given the context-dependent nature of antioxidant regulation and the well-documented sex-dependent molecular heterogeneity of MDD, we also performed an exploratory diagnosis-by-sex analysis, with full results presented in Supplementary Table S2. Previous postmortem transcriptomic studies have identified largely distinct and, in some cases, oppositely directed transcriptional alterations in men and women with MDD, including in the dlPFC [62,63].

*GPX4* emerged as the most notable example of potentially sex-divergent redox regulation. *GPX4* encodes glutathione peroxidase 4, a glutathione-dependent enzyme that reduces phospholipid hydroperoxides, limits membrane lipid peroxidation, and protects cells against ferroptosis. Disruption of the GPX4-dependent ferroptosis–mitochondrial axis has been proposed to contribute to oxidative stress, metabolic dysfunction, and neuroinflammation in depression [20]. In the exploratory analysis, *GPX4* expression was significantly higher in men with MDD after panel-level correction, whereas the corresponding effect in women was weakly negative. However, the diagnosis-by-sex interaction was only nominally significant and did not survive correction across the 14-gene panel; this pattern should therefore be regarded as hypothesis-generating rather than as a confirmed sex-specific effect. This finding is directionally consistent with an independent postmortem study in which *GPX4* was identified as a hub gene within a mitochondria-enriched co-expression module in the nucleus accumbens that was enriched for transcripts upregulated in men, but not women, with MDD [64]. The module showed opposite-direction relationships with insomnia/hypersomnia in men and women, although only the association in women survived correction. Because that finding was derived from a different brain region and a network-based analytical framework, it does not constitute a direct replication of the present dlPFC result but supports a sex-dependent context for *GPX4* regulation in MDD.

*MTOR* showed another potentially male-associated pattern, with lower expression in men with MDD but no significant diagnosis-by-sex interaction. mTOR coordinates activity-dependent translation, synaptic protein synthesis, cellular metabolism, and adaptive stress responses. Reduced levels of mTOR and downstream translational-signaling proteins, including p70S6K and eIF4B, have previously been reported in the prefrontal cortex of individuals with MDD [65]. Together, the *GPX4* and *MTOR* findings support potentially sex-modulated alterations in lipid-redox, mitochondrial, metabolic, and synaptic stress-response pathways rather than a uniform antioxidant deficit.

### 4.6. Cross-Cohort Reproducibility and Cell-Type Context

The main strength of the present analysis lies in its emphasis on reproducibility across heterogeneous postmortem datasets. Agreement between the primary Fisher analysis, sample-size-weighted signed Stouffer analysis, one-stage generalized least-squares models, random-effects meta-analysis, and permutation testing strengthens support for the five-gene pattern. The absence of diagnosis-by-cohort interactions after multiple-testing correction further indicates that no single cohort showed statistically robust evidence of a distinct diagnostic effect after accounting for the panel-wide testing burden.

The permutation analysis provides an additional panel-level assessment of specificity. None of the 200,000 disease-label permutations produced at least five genes meeting the complete primary-support criterion. This result indicates that the observed combination of aggregate statistical evidence and directional concordance was highly unlikely under random reassignment of diagnostic labels. Nevertheless, permutation support does not rule out residual confounding related to cohort composition, tissue annotation, medication exposure, postmortem factors, platform, or preprocessing.

The descriptive single-nucleus analysis placed *NPTX2*, *BDNF*, *VGF*, and *EGR1* predominantly in a neuronal detection context, whereas *SAT1* showed a broader cellular distribution. These findings provide biological localization but do not establish cell-type-specific differential expression. The observed distributions may reflect expression enrichment, differences in cell abundance, transcript-detection probability, or a combination of these factors. Because the primary analyses were based largely on bulk tissue, lower expression of *BDNF*, *VGF*, *EGR1*, *NPTX2*, and *SAT1* may reflect not only cell-intrinsic transcriptional suppression but also altered neuronal and glial representation within the dlPFC tissue. Postmortem morphometric studies have demonstrated reduced neuronal size and glial density in area 9, as well as broader reductions in the size and density of neurons and glial cells across dlPFC cortical layers in MDD [66,67]. The predominantly neuronal localization observed in GSE213982 identifies the likely cellular context of these genes but cannot distinguish transcriptional regulation from structural or compositional changes. Cell-type-resolved, spatial, and morphometric analyses will therefore be required to clarify the origin of the bulk-tissue signal.

### 4.7. Functional and Cognitive Relevance of the dlPFC

The shared lower-expression direction of *BDNF*, *VGF*, *EGR1*, *NPTX2*, and *SAT1* in the dlPFC supports the interpretation that MDD may be associated with reduced molecular capacity for adaptive plasticity and stress-related regulation [11,17,31]. This pattern encompassed neurotrophic support, activity-dependent transcription, synaptic organization, and polyamine metabolism rather than a single isolated pathway. Its regional localization is important because peripheral molecular markers cannot determine whether an alteration involves prefrontal, limbic, hippocampal, striatal, or other circuits implicated in depression. Postmortem transcriptomic analysis provides a distinct level of evidence by linking molecular alterations directly to an anatomically defined cortical region. The dlPFC is strongly involved in “cold” executive functions, including working memory, attention, planning, inhibitory control, cognitive flexibility, and goal-directed regulation, whereas more ventromedial prefrontal regions are more closely associated with affective valuation, reward processing, and socio-emotional integration [7,68,69,70,71]. The dlPFC-centered expression pattern observed here may therefore be particularly relevant to impaired cognitive control, reduced flexibility, and deficient updating of internal representations in MDD. Such impairment may contribute to the persistence of maladaptive self-referential beliefs described in cognitive models of depression, including Beck’s cognitive triad [72,73]. However, this interpretation remains indirect, as the present transcriptomic findings do not constitute direct measures of executive function, cognition, or symptom content.

### 4.8. Limitations

Several limitations should be noted. The cohorts differed in sample composition, clinical annotation, medication exposure, postmortem variables, platform, preprocessing, and covariate availability, preventing a fully harmonized adjusted analysis and leaving the possibility of residual confounding. Because most analyses were based on bulk tissue, the observed differences may reflect both cell-intrinsic regulation and variation in cellular composition; the single-nucleus analysis provided only descriptive localization.

Transcript levels do not directly indicate protein abundance, enzymatic activity, synaptic function, or cellular redox state. Accordingly, the present findings should not be interpreted as direct evidence of increased oxidative damage, impaired antioxidant capacity, or altered ferroptotic activity, as ROS, glutathione status, lipid-peroxidation products, polyamine levels, and antioxidant-enzyme activities were not measured.

The restricted 14-gene panel does not represent the full MDD transcriptome. In addition, GSE54567 and GSE54568 comprised distinct matched sample sets but originated from the same parent study and GPL570 platform and therefore were not fully independent study-level or technical replications. The paired analyses accounted for case–control matching within each cohort but did not remove the shared project- and platform-level context. Finally, the cross-sectional postmortem design does not allow causal inference or distinguish disease-related changes from treatment effects, chronic illness, or compensatory responses.

## 5. Conclusions

Canonical antioxidant-enzyme transcripts did not show consistent cross-cohort support, arguing against a generalized antioxidant deficit or a stable oxidative-stress transcriptomic signature in MDD. Their discordant expression patterns may instead reflect heterogeneous, context-dependent adaptive or compensatory responses to chronic cellular stress, although this interpretation cannot be established from the present postmortem bulk-tissue data. Across seven postmortem dlPFC cohorts, MDD was associated with a reproducible lower-expression pattern linking redox-sensitive stress adaptation with neurotrophic signaling, activity-dependent transcription, synaptic organization, and polyamine metabolism. The convergence of *NPTX2*, *EGR1*, *VGF*, *BDNF*, and *SAT1* is consistent with reduced molecular capacity for neuronal resilience and adaptive plasticity in MDD. These findings require functional and cell-type-specific validation.

## Figures and Tables

**Table 1:** **Public postmortem prefrontal cortex datasets included in the primary** **analysis.**

Dataset	Brain Region	Data Type	MDD Cases	Controls
GSE54567	dlPFC	Microarray	14	14
GSE54568	dlPFC	Microarray	15	15
GSE102556	dlPFC	RNA-seq	26	22
GSE101521	dlPFC	RNA-seq	30	29
GSE53987	dlPFC	Microarray	17	19
GSE208338	dlPFC	Microarray	24	62
GSE213982	dlPFC	RNA-seq	20	18
**Total**	**—**	**—**	**146**	**179**

**Table 2:** **Primary cross-cohort results for the 14-gene candidate panel.** The table reports Fisher-combined *p*-values, Benjamini–Hochberg-adjusted values, direction patterns, and primary-support status. Direction patterns follow the order GSE54567, GSE54568, GSE102556, GSE101521, GSE53987, GSE208338, and GSE213982. *SAT1* was unavailable in the processed GSE208338 matrix; therefore, its direction pattern includes six cohorts. Upward arrows (↑) indicate higher expression in MDD, whereas downward arrows (↓) indicate lower expression in MDD.

Gene	Direction Pattern	Fisher *p*	Fisher BH-FDR	Primary Support
*EGR1*	↓ ↓ ↓ ↓ ↓ ↓ ↓	5.01 × 10^−5^	0.000701	Yes
*NPTX2*	↓ ↓ ↓ ↓ ↓ ↓ ↓	0.000168	0.00118	Yes
*VGF*	↓ ↓ ↓ ↓ ↓ ↓ ↓	0.000579	0.00270	Yes
*BDNF*	↓ ↓ ↓ ↓ ↓ ↓ ↓	0.0114	0.0337	Yes
*SAT1*	↓ ↓ ↓ ↓ ↓ ↓	0.0127	0.0337	Yes
*GPX4*	↑ ↓ ↑ ↓ ↑ ↓ ↑	0.0145	0.0337	No
*SKA2*	↓ ↓ ↓ ↑ ↓ ↓ ↓	0.0254	0.0507	No
*ADAMTS16*	↓ ↓ ↓ ↓ ↑ ↓ ↑	0.0449	0.0725	No
*PRDX2*	↑ ↑ ↑ ↓ ↑ ↓ ↑	0.0466	0.0725	No
*SOD2*	↑ ↑ ↑ ↓ ↑ ↓ ↓	0.0541	0.0757	No
*MTOR*	↓ ↑ ↓ ↓ ↑ ↓ ↓	0.1109	0.141	No
*PRDX3*	↑ ↓ ↑ ↓ ↑ ↓ ↓	0.145	0.169	No
*MFN1*	↓ ↓ ↓ ↓ ↑ ↓ ↓	0.184	0.198	No
*ZNF701*	↑ ↑ ↑ ↑ ↑ ↓ ↑	0.552	0.552	No

**Table 3:** **One-stage individual-level sensitivity analysis across seven analytical cohorts.** Negative coefficients indicate lower expression in MDD. Models included cohort-specific intercepts and covariance blocks accounting for matched pairs in GSE54567 and GSE54568. Expression was standardized within each gene and cohort. Benjamini–Hochberg correction was applied across the prespecified 14-gene panel. *SAT1* included six cohorts because processed expression values were unavailable in GSE208338.

Gene	Standardized Diagnosis β	95% CI	*p*-Value	BH-FDR	Diagnosis × Cohort *p*-Value
*NPTX2*	−0.554	−0.753 to −0.354	9.16 × 10^−8^	1.28 × 10^−6^	0.822
*EGR1*	−0.557	−0.769 to −0.346	3.83 × 10^−7^	2.68 × 10^−6^	0.503
*VGF*	−0.483	−0.694 to −0.273	8.76 × 10^−6^	4.09 × 10^−5^	0.176
*BDNF*	−0.432	−0.624 to −0.241	1.21 × 10^−5^	4.23 × 10^−5^	0.966
*SAT1*	−0.435	−0.671 to −0.199	0.000351	0.000983	0.555

**Table 4:** **Descriptive cell-type localization of the five primary-support genes in the GSE213982 single-nucleus RNA-seq dlPFC dataset.** Percentages represent the proportion of total observed gene counts assigned to excitatory and inhibitory neuronal nuclei. These values were used only to provide cellular context and were not interpreted as cell-type-specific differential expression.

Gene	% Observed Counts in Neuronal Nuclei, ExN + InN
*NPTX2*	96.0%
*BDNF*	96.9%
*VGF*	95.7%
*EGR1*	91.5%
*SAT1*	75.9%

## Data Availability

All datasets analyzed in this study are publicly available from the Gene Expression Omnibus under accession numbers GSE54567, GSE54568, GSE102556, GSE101521, GSE53987, GSE208338, and GSE213982. The analyses used publicly deposited series matrices, processed expression tables, and count matrices, as described in the Materials and Methods. The prespecified 14-gene panel and its biological-domain structure were fixed and documented in a time-stamped OSF project created on 29 May 2026, before the primary cross-cohort statistical analyses were conducted. The documentation is available at https://osf.io/8bfrs/overview?view_only=6f7fbb64f2da46ada7fd619c72a7d86d (accessed on 30 May 2026) Analysis code and processed input files required to reproduce the cohort-level tests, Fisher and sample-size-weighted signed Stouffer analyses, Benjamini–Hochberg correction, one-stage generalized least-squares analysis, random-effects meta-analysis, Shapiro–Wilk diagnostics, probe-level sensitivity analyses, exploratory diagnosis-by-sex analysis, descriptive cell-type localization, permutation analysis, and figure and table generation are provided in Supplementary File S1. FASTQ- and CEL-level reprocessing was not performed.

## Supplementary Materials

The following supporting information can be downloaded at: https://www.mdpi.com/article/10.3390/antiox15070908/s1, Supplementary Table S1: Sample-level expression data and sex assignments; Supplementary Table S2: Summary of the primary, sensitivity, and exploratory sex-stratified analyses; Supplementary File S1: Complete reproducibility package containing processed input data, analysis scripts, result files, validation tools, the 200,000-permutation analysis report, and the corresponding computational code.
