# Supplementary material for: Convergent Lower Expression of Redox-Linked Stress-Adaptation and Synaptic-Plasticity Genes in Major Depressive Disorder Across Seven Postmortem dlPFC Cohorts
Source: Antioxidants (Basel). 2026 Jul 22;15(7):908. doi: 10.3390/antiox15070908 (PMC13405805; doi:10.3390/antiox15070908)
Supplement: Supplementary file 1 [file antioxidants-15-00908-s001.zip › Supplementary_File_S1_reproducibility_package_7cohort_with_Hedges/reports/permutation_sensitivity_200k.pdf]

Permutation-Based Sensitivity Analysis

Fixed 14-gene MDD postmortem DLPFC panel across seven analytical cohorts

Disease labels were permuted independently within each cohort while preserving the original study design. For GSE54567 and GSE54568, diagnosis was permuted by random case-control swaps within matched pairs. For GSE102556, GSE101521, GSE53987, GSE208338, and GSE213982, diagnosis labels were permuted while preserving the observed group sizes. The same permuted labels were applied to all 14 genes within a cohort to preserve their correlation structure. For each of 200,000 permutations, cohort-level paired or Welch tests were repeated, p-values were combined using Fisher's method, Benjamini-Hochberg correction was applied across the fixed panel, and concordant direction across all available cohorts was required. Empirical p-values used add-one correction. Random seed: 20260625.

Quantity	Observed / estimate
Observed primary-support genes	5
Genes	NPTX2, EGR1, VGF, BDNF, SAT1
Permutations	200,000
Permutations with >= observed count	0
Panel-level empirical p	4.999975e-06

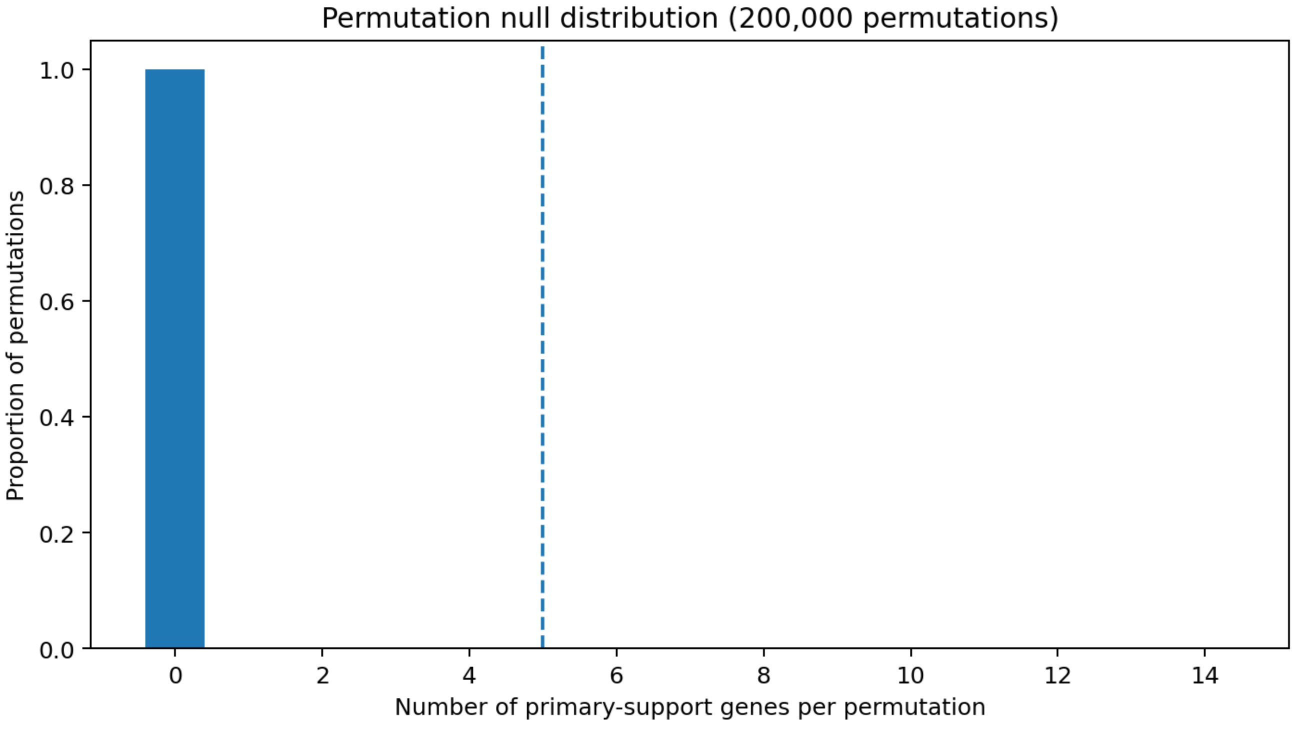

The empirical panel-level p-value quantifies how often the complete primary pipeline produced at least 5 primary-support genes under randomized diagnosis labels.

Gene-level observed and permutation results

Gene	K	Fisher p	BH-FDR	Direction	Primary support	Permutation frequency
GPX4	7	0.01446	0.03375	discordant	No	0.00012
PRDX2	7	0.0466	0.07249	discordant	No	9e-05
VGF	7	0.0005792	0.002703	7/7 down	Yes	7e-05
ADAMTS16	7	0.04489	0.07249	discordant	No	6.5e-05
NPTX2	7	0.0001684	0.001179	7/7 down	Yes	7.5e-05
EGR1	7	5.008e-05	0.0007011	7/7 down	Yes	5.5e-05
ZNF701	7	0.5521	0.5521	discordant	No	0.0001
MFN1	7	0.184	0.1981	discordant	No	7.5e-05
SOD2	7	0.05411	0.07575	discordant	No	8e-05
MTOR	7	0.1109	0.1411	discordant	No	8.5e-05
PRDX3	7	0.145	0.1692	discordant	No	4e-05
SKA2	7	0.02537	0.05073	discordant	No	8e-05
SAT1	6	0.01267	0.03375	6/6 down	Yes	0.00013
BDNF	7	0.01141	0.03375	7/7 down	Yes	6.5e-05

Permutation frequency is the add-one corrected proportion of permutations in which the gene itself met the full primary-support rule (BH-FDR < 0.05 and concordant direction). It is descriptive and is not used to redefine the primary analysis.
