# Supplementary figures and images for: Convergent Lower Expression of Redox-Linked Stress-Adaptation and Synaptic-Plasticity Genes in Major Depressive Disorder Across Seven Postmortem dlPFC Cohorts
Source: Antioxidants (Basel). 2026 Jul 22;15(7):908. doi: 10.3390/antiox15070908 (PMC13405805; doi:10.3390/antiox15070908)

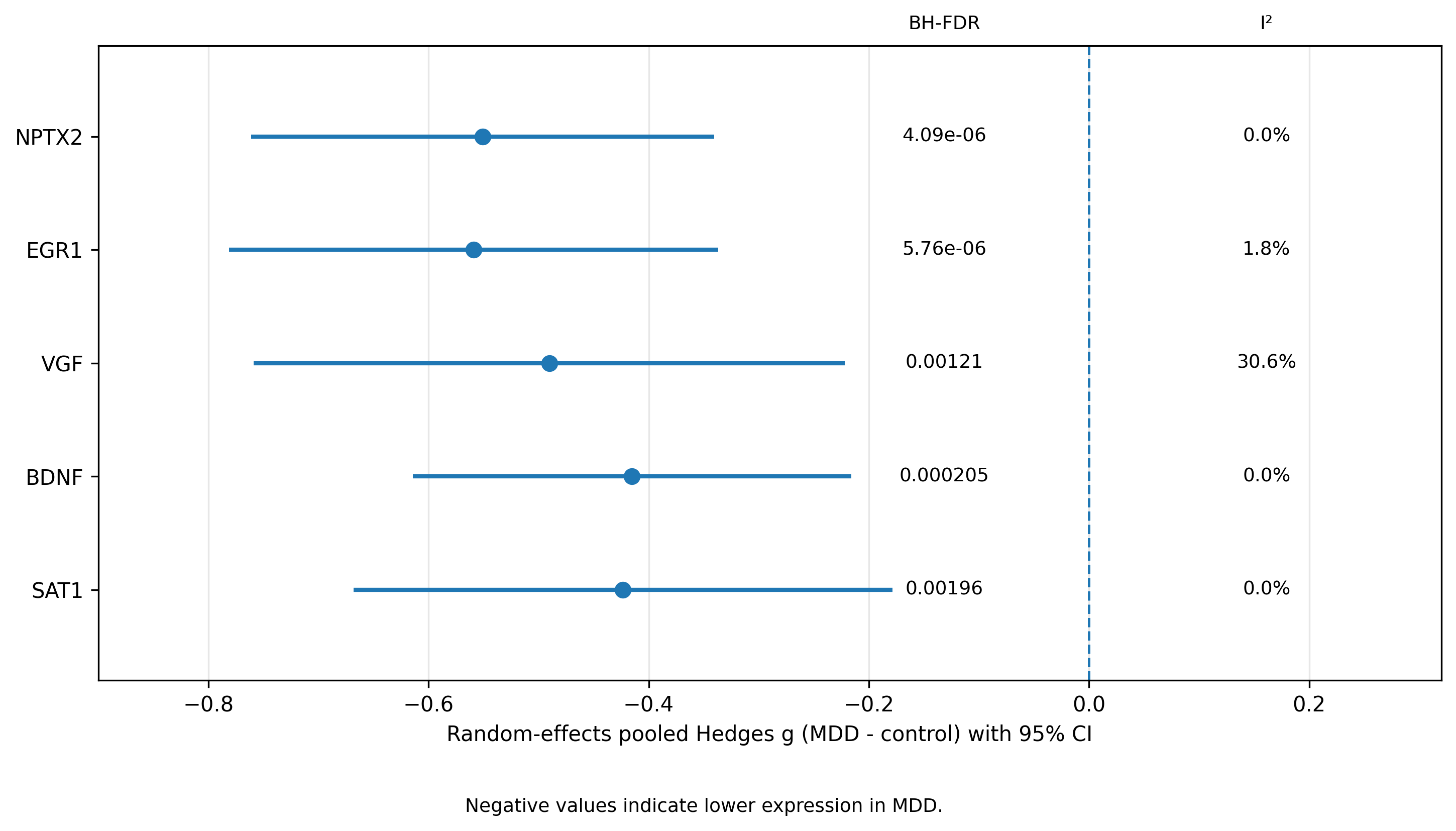

Supplement: Supplementary file 1 [file antioxidants-15-00908-s001.zip › Supplementary_File_S1_reproducibility_package_7cohort_with_Hedges/reports/Hedges_g_primary_genes_forest_plot.png]
